# Dysregulation of Retinal Melatonin Biosynthetic Pathway and Differential Expression of Retina-Specific Genes Following Blast-Induced Ocular Injury in Ferrets

**DOI:** 10.3390/neurolint17030042

**Published:** 2025-03-17

**Authors:** Chetan Pundkar, Rex Jeya Rajkumar Samdavid Thanapaul, Manoj Govindarajulu, Gaurav Phuyal, Joseph B. Long, Peethambaran Arun

**Affiliations:** 1 Blast-Induced Neurotrauma Branch, Center for Military Psychiatry and Neuroscience, Walter Reed Army Institute of Research, Silver Spring, MD 20910, USA; chetan.y.pundkar.ctr@health.mil (C.P.); rexjeyarajkumar.samdavidthanapaul.ctr@health.mil (R.J.R.S.T.); manoj.y.govindarajulu.ctr@health.mil (M.G.); gaurav.phuyal.ctr@health.mil (G.P.); joseph.b.long.civ@health.mil (J.B.L.); 2 National Research Council (NRC) Research Associateship Programs, National Academies of Sciences, Engineering, and Medicine, Washington, DC 20001, USA

**Keywords:** blast-induced traumatic ocular injury, retina, melatonin

## Abstract

Background/Objectives: Blast-induced traumatic ocular injuries (bTOI) pose a significant risk to military and civilian populations, often leading to visual impairment or blindness. Retina, the innermost layer of ocular tissue consisting of photoreceptor and glial cells, is highly susceptible to blast injuries. Despite its prevalence, the molecular mechanisms underlying retinal damage following bTOI remain poorly understood, hindering the development of targeted therapies. Melatonin, a neuroprotective indoleamine with antioxidant, anti-inflammatory, and circadian regulatory properties, is synthesized in the retina and plays a crucial role in retinal health. Similarly, retina-specific genes, such as *Rhodopsin*, *Melanopsin*, and RPE65, are essential for photoreceptor function, visual signaling, and the visual cycle. However, their responses to blast exposure have not been thoroughly investigated. Methods: In this study, we utilized a ferret model of bTOI to evaluate the temporal expression of melatonin-synthesizing enzymes, such as tryptophan hydroxylase 1 and 2 (*TPH*1 and *TPH*2), Aralkylamine N-acetyltransferase (*AANAT*), and Acetylserotonin-O-methyltransferase (*ASMT*), and retina-specific genes (*Rhodopsin*, *Melanopsin*) and retinal pigment epithelium-specific 65 kDa protein (*RPE65*) at 4 h, 24 h, 7 days, and 28 days post-blast. Ferrets were exposed to tightly coupled blast overpressure waves using an advanced blast simulator, and retinal tissues were collected for quantitative polymerase chain reaction (qPCR) analysis. Results: The results revealed dynamic and multiphasic transcriptional responses. *TPH*1 and *TPH*2 exhibited significant upregulation at 24 h, followed by downregulation at 28 days, indicating blast-induced dysregulation of tryptophan metabolism, including melatonin synthesis. Similarly, *AANAT* and *ASMT* showed acute downregulation post-blast, with late-phase disruptions. *Rhodopsin* expression increased at 24 h but declined at 28 days, while *Melanopsin* and *RPE65* demonstrated early upregulation followed by downregulation, reflecting potential disruptions in circadian regulation and the visual cycle. Conclusions: These findings highlight the complex regulatory mechanisms underlying retinal responses to bTOI, involving neuroinflammation, oxidative stress, and disruptions in melatonin synthesis and photoreceptor cell functions. The results emphasize the therapeutic potential of melatonin in mitigating retinal damage and preserving visual function.

## 1. Introduction

Blast-induced traumatic ocular injuries (bTOI) pose a serious risk to both military and civilian populations, and, in both, loss of vision can disrupt careers and adversely impact lifestyle [1]. Among military personnel, eye injuries rank as the fourth most prevalent battlefield injury, constituting 6–13% of all blast injuries [2]. In particular, explosions during combat significantly contribute to ocular injuries, which often result in visual impairment or permanent blindness [3,4]. The incidence and severity of these ocular injuries have been exacerbated by the advancement of weaponry with enhanced explosive capabilities [5]. Ocular trauma, primarily due to blast injuries from improvised explosive devices (IEDs), has been a leading cause of injury among U.S. service members [6]. The bTOI can be primary, secondary, tertiary, or quaternary, resulting from various sources such as blast waves, fragments, structural collapse, burns, and indirect injuries, respectively [7]. Bilateral ocular explosion injuries are common [8]. The blast impacts numerous regions throughout the eye such as eyelids, cornea, conjunctiva, retina, and optic nerve [3]. Increased light sensitivity, retinal separation, retinal edema, retinopathy, optic neuropathy, and loss of visual field are among the clinical consequences of bTOI [9]. The retina is highly susceptible to shockwaves resulting from blasts. However, the lack of understanding of the molecular mechanisms underlying retinal damage post bTOI hinders the development of targeted therapies.

Melatonin, an indoleamine molecule produced by the pineal gland from the amino acid tryptophan, regulates circadian rhythms [10]. The first and rate-limiting step for synthesizing the neurotransmitter serotonin from tryptophan is catalyzed by *TPH*1 and *TPH*2. This hydroxylation of tryptophan, followed by decarboxylation, results in the formation of 5-hydroxytryptamine (serotonin). *AANAT* converts serotonin to N-acetylserotonin, which is considered the rate-limiting step in synthesizing melatonin, after which ASMT catalytically converts N-acetylserotonin to melatonin [11]. Melatonin is also synthesized from tryptophan in the retina, skin, kidneys, gastrointestinal tract, and spleen through the same metabolic pathway [12]. Along with regulating circadian cycles, melatonin also plays a vital role in immunological, cardiovascular, and retinal functioning [13]. It has the potential to be a therapeutic and neuroprotective agent in neurodegenerative and age-related disorders due to its antioxidant properties and ability to protect against oxidative stress [14]. Melatonin acts via receptors that mediate its essential role in ocular angiogenesis and other significant effects on the immune system, peripheral organs, and the central nervous system. Previous studies have shown the salutary anti-inflammatory and antioxidant properties of melatonin in retinal diseases such as age-related macular degeneration (AMD) and diabetic retinopathy (DR) [15]. However, the role of melatonin has not yet been explored in the pathogenesis of bTOI.

Retina-associated photoreceptors, pigments, and enzymes play a crucial role in normal vision. *Melanopsin*, a photopigment in intrinsically photosensitive retinal ganglion cells (ipRGCs), regulates melatonin synthesis in the retina [16]. When activated by light, it provides information about ambient light levels to the brain, regulating the pineal gland’s melatonin production [16] and synchronizing the body’s circadian cycles with the external light–dark cycle. *RPE65* maintains photoreceptors’ responsiveness to light, thereby contributing to signaling cascades involving *Melanopsin*-expressing ipRGCs [17]. *Rhodopsin*, a light-sensitive receptor protein in the retinal photoreceptive rod cells, is necessary for vision in low-light environments and helps control the body’s circadian cycles [18]. Alterations in the time-dependent expression pattern of these retina-specific genes in blast-induced retinal injury have not been previously described.

This study explored the temporal expression pattern of genes involved in retinal melatonin production and retina-specific genes in the retina after blast exposure in ferrets.

## 2. Materials and Methods

### 2.1. Animals

Adult male ferrets (Mustela putorius furo), aged 13–15 weeks and weighing around 1–1.2 kg, were purchased from Triple F Farms (Gillett, PA, USA) and were socially housed in pairs in ventilated cages at 20–22 °C on a 12:12 h light–dark cycle. Ferrets were provided with free access to food and chlorinated water ad libitum throughout the course of the study. Research using ferrets was conducted under an Institutional Animal Care and Use Committee-approved animal use protocol in an AAALAC International-accredited facility in compliance with the Animal Welfare Act and other federal statutes and regulations relating to animals and experiments involving animals and adhered to principles stated in the Guide for the Care and Use of Laboratory Animals, NRC Publication, 2011 edition. Ferrets were randomized into two groups: sham and repeated blast (BB), with each group containing a minimum of four ferrets.

### 2.2. Blast Exposure and Sample Collection

Ferrets were anesthetized with 5% isoflurane for 8 min and positioned in a longitudinal prone orientation (facing the oncoming shockwave) within the test section of an advanced blast simulator (ABS) [19]. Each ferret was exposed to two 19 psi blast overpressure waves, separated by a 2 min interval as described earlier [19]. Following blast exposure, the ferrets were euthanized at 4 h, 24 h, 7 days, or 28 days. Retinas from both eyes were dissected as described previously [20]. Retinal dissection involves an incision at the corneoscleral junction and removing the anterior part of the eye, including the cornea and lens. The short stalk of the optic nerve on the posterior side of the eye was secured with tweezers, and the eyeball was gently squeezed in the opposite direction to allow the retina to float out of the sclera. Retinas from both eyes were pooled and preserved in phosphate-buffered saline (PBS) and stored at −80 °C for further analysis.

### 2.3. RNA Extraction and Quantitative RT-PCR

Total RNA was extracted from the retina using the RNeasy Lipid Tissue Mini Kit (Cat #74104, Qiagen, Redwood City, CA, USA). RNA quantity was measured using the Nanodrop D2000 spectrophotometer (Thermo Scientific, Wilmington, DE, USA), and sample purity was assessed by the absorbance ratio at 260/280 nm. Only samples with a ratio of 1.8–2.0 were used for cDNA synthesis. RNA was reverse transcribed into cDNA using the RT2 First Strand Kit (Cat #330404, Qiagen, MD, USA), and the cDNA was stored at −20 °C until further use. Primers used in the study were procured from Eurofins Genomics (Louisville, KY, USA) and are listed in Table 1. Quantitative real-time polymerase chain reaction (qRT-PCR) was performed on the synthesized cDNA using RT2 SYBR Green qPCR Mastermix reagent, followed by amplification on an Applied QuantStudio 6 Flex qPCR system (Life Technologies, Grand Island, NY, USA). Each sample was run in triplicate, and the average critical threshold cycle (Ct) was used to calculate relative quantification by fold change and statistical significance. No-template controls were included in triplicate for each probe in every run to ensure accuracy and prevent contamination. Relative mRNA expression was normalized to the housekeeping gene *18S rRNA*, and the fold changes were calculated using the 2^−ΔΔCt^ method. The ΔΔCt calculation involved determining the difference between the ΔCt values of the blast group and the mean ΔCt value of the sham group. Fold changes in specific mRNA expression in the blast group relative to the sham group were presented as normalized fold changes.

## 3. Statistical Analysis

All data are expressed as the mean ± SEM and were compared using a student *t*-test to assess the effects of blast exposure compared to the sham group. Statistical analyses were conducted using GraphPad Prism 9 software (GraphPad Software Inc., Boston, MA, USA). Differences among the groups were considered statistically significant at *p*-values < 0.05.

## 4. Results

### 4.1. Effect of Blast Exposure on Expression of Melatonin-Synthesizing Enzymes

**Figure 1:**
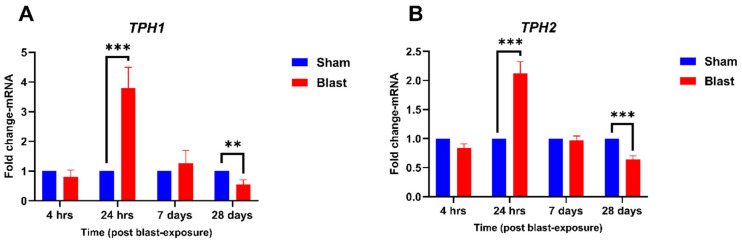
Temporal expression of *TPH*1 and *TPH*2 mRNA in the retina following blast exposure. Fold changes in mRNA levels of *TPH*1 and *TPH*2 were quantified at 4 h, 24 h, 7 days, and 28 days post-blast exposure, normalized to sham controls. (**A**) *TPH*1 mRNA expression showed a biphasic pattern, with significant upregulation at 24 h (*p* < 0.001) and downregulation at 28 days (*p* < 0.01). (**B**) *TPH*2 mRNA expression exhibited a similar biphasic trend, with significant upregulation at 24 h (*p* < 0.001) and downregulation at 28 days (*p* < 0.001). Data are presented as mean ± SEM, with statistical significance indicated as, ** *p* < 0.01, and *** *p* < 0.001.

*TPH* exists in two isoforms, *TPH*1 and *TPH*2, with *TPH*2 being primarily expressed in the brain and retina, while *TPH1* is mainly found in the gut and pineal gland [21]. In the retina, melatonin is predominantly synthesized by the photoreceptor cells and other retinal cell types during certain pathological conditions [22]. Studies have shown that *TPH2* expression in the retina exhibits a circadian rhythm, suggesting a potential role in regulating the body’s internal clock [23,24]. To explore the impact of blast exposure on the melatonin synthesis pathway, we investigated the mRNA expression levels of *TPH1* and *TPH2* in the retina at various time points. The temporal expression of ***TPH*1** and ***TPH*2 mRNA** in the retina following blast exposure was assessed at four time points (4 h, 24 h, 7 days, and 28 days). Both enzymes exhibited dynamic and biphasic expression patterns. For ***TPH*1** (Figure 1A), no significant difference in mRNA expression was observed between the blast and sham groups at 4 h post-blast (*p* > 0.05). At 24 h, *TPH1* mRNA expression was significantly upregulated, with a fold change of 3.80 compared to the sham group (*p* < 0.001), representing the peak expression level. By 7 days, *TPH1* mRNA levels returned to baseline, showing no significant difference from sham controls. At 28 days, *TPH1* mRNA expression was significantly downregulated compared to the sham group, with a fold change of approximately 0.5 (*p* < 0.01). Similarly, ***TPH*2 mRNA** followed a comparable temporal pattern (Figure 1B), with no significant changes observed at 4 h. At 24 h, *TPH*2 mRNA was significantly upregulated, showing a fold change of 2.12 (*p* < 0.001). By 7 days, *TPH*2 expression normalized to baseline, while at 28 days, it was significantly downregulated, with a fold change of approximately 0.63 (*p* < 0.001). These findings suggest that *TPH*1 and *TPH*2 are involved in acute and long-term retinal responses to blast injury, including neuroinflammation, oxidative stress, and neuroplasticity, due to potential dysregulation of melatonin synthesis, as shown below.

**Figure 2:**
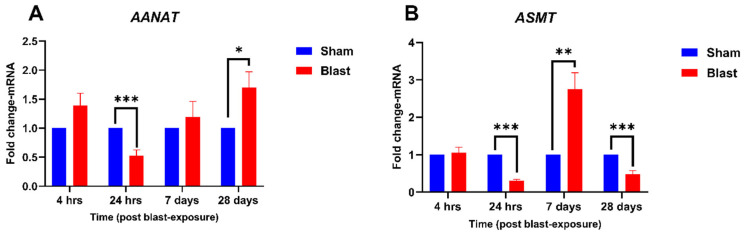
Temporal expression of *AANAT* and *ASMT* mRNA in the retina following blast exposure. Fold changes in *AANAT* and *ASMT* mRNA levels were quantified at 4 h, 24 h, 7 days, and 28 days post-blast exposure, normalized to sham controls. (**A**) *AANAT* mRNA expression demonstrated significant downregulation at 24 h (*p* < 0.001) and upregulation at 28 days (*p* < 0.05). (**B**) *ASMT* mRNA expression showed dynamic regulation, with significant downregulation at 24 h (*p* < 0.001), upregulation at 7 days (*p* < 0.01), and downregulation again at 28 days (*p* < 0.001). Data are presented as mean ± SEM, with statistical significance indicated as * *p* < 0.05, ** *p* < 0.01, and *** *p* < 0.001.

To explore the impact of blast exposure on melatonin synthesis, we further investigated the mRNA expression of *AANAT* and *ASMT* in the retina. The expression of ***AANAT*** and ***ASMT* mRNA**, key enzymes in melatonin synthesis, was also analyzed, revealing dynamic multiphasic responses to blast exposure. For ***AANAT*** (Figure 2A), expression at 4 h post-blast showed a non-significant upward trend compared to the sham group. At 24 h, *AANAT* mRNA expression was significantly downregulated (*p* < 0.001), reflecting an acute inhibitory effect of blast injury on melatonin synthesis. By 7 days, expression returned to baseline, showing no significant difference from sham controls. At 28 days, *AANAT* mRNA was significantly upregulated compared to the sham group (*p* < 0.05), with a fold change of 1.69, suggesting a late-phase compensatory activation for the decreased expression of *TPH*1&2 genes. For ***ASMT*** (Figure 2B), no significant differences were observed at 4 h post-blast. At 24 h, *ASMT* expression was significantly downregulated (*p* < 0.001), while at 7 days, a substantial upregulation with a fold change of 2.74 was observed (*p* < 0.01). By 28 days, *ASMT* mRNA was significantly downregulated again, showing a fold change of 0.47 (*p* < 0.001). These findings suggest blast exposure affects melatonin synthesis through complex regulatory mechanisms involving *AANAT* and *ASMT*, with implications for circadian signaling and retinal neuroprotection.

### 4.2. Effect of Blast Exposure on Expression of Retina-Specific Genes

Blast exposure can lead to disturbances in vision by disrupting the function of retinal cells [25,26]. However, direct research on the interaction between certain retina-specific proteins such as *Rhodopsin*, *Melanopsin*, and *RPE65* and blast exposure is limited. Therefore, we investigated the effects of repeated blast exposure on the mRNA expression of these key proteins at various time points post-blast.

**Figure 3:**
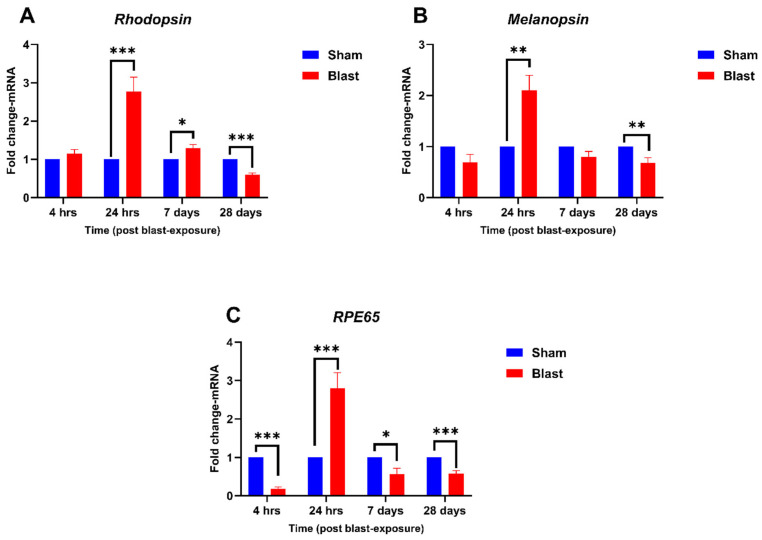
Temporal expression of *Rhodopsin*, *Melanopsin*, and *RPE65* mRNA in the retina following blast exposure. Fold changes in mRNA levels of *Rhodopsin, Melanopsin*, and *RPE65* were quantified at 4 h, 24 h, 7 days, and 28 days post-blast exposure, normalized to sham controls. (**A**) *Rhodopsin* mRNA expression showed a biphasic pattern with significant upregulation at 24 h (*p* < 0.001), and 7 days (*p* < 0.05), and significant downregulation at 28 days (*p* < 0.001). (**B**) *Melanopsin* mRNA expression also exhibited a biphasic change with significant upregulation at 24 h (*p* < 0.01) and downregulation at 28 days (*p* < 0.01). (**C**) *RPE65* mRNA expression demonstrated a triphasic change with significant downregulation at 4 h (*p* < 0.001), upregulation at 24 h (*p* < 0.001), and downregulation at 7 days (*p* < 0.05) and 28 days (*p* < 0.001). Data are presented as mean ± SEM, with statistical significance indicated as * *p* < 0.05, ** *p* < 0.01, and *** *p* < 0.001.

Retina-specific genes, ***Rhodopsin***, ***Melanopsin***, and ***RPE65,*** exhibited distinct transcriptional responses to blast exposure, reflecting potential disruptions in photoreceptor function, retinal signaling, and visual cycle maintenance post-blast. For ***Rhodopsin*** (Figure 3A), expression remained stable at 4 h post-blast. At 24 h, significant upregulation was observed, with a fold change of 2.76 compared to the sham group (*p* < 0.001). This upregulation persisted at 7 days, albeit at a lower level, with a fold change of 1.28 (*p* < 0.05). At 28 days, *Rhodopsin* expression was significantly downregulated (*p* < 0.001), with a fold change of 0.60, suggesting long-term disruptions in photoreceptor function. ***Melanopsin*** expression (Figure 3B) showed no significant changes at 4 h. At 24 h, *Melanopsin* mRNA was significantly upregulated with a fold change of 2.10 (*p* < 0.01), while at 7 days, it normalized to baseline. At 28 days, *Melanopsin mRNA* was significantly downregulated, with a fold change of 0.67 (*p* < 0.01), indicating potential long-term alterations in circadian regulation. ***RPE65***, essential for the visual cycle, exhibited (Figure 3C) an acute downregulation at 4 h (*p* < 0.001). At 24 h, a marked upregulation of *RPE65* mRNA was observed, with a fold change of 2.79 (*p* < 0.001), potentially reflecting a compensatory or inflammatory response. By 7 days, *RPE65* expression declined (*p* < 0.05), and at 28 days, it was downregulated further, showing a fold change of approximately 0.58 (*p* < 0.001). These results suggest that *Rhodopsin*, *Melanopsin*, and *RPE65* are integral to the retinal response to blast injury, with dynamic transcriptional changes indicating acute, sub-acute, and chronic effects.

Collectively, these findings demonstrate that blast exposure induces distinct temporal changes in the transcription of melatonin-synthesizing enzymes and retina-specific genes, highlighting their roles in neuroinflammation, oxidative stress, circadian signaling, and visual function. The multiphasic dysregulation observed across these genes underscores the complexity of retinal responses to blast injury and the need for further studies to elucidate their functional significance.

## 5. Discussion

The retina is highly susceptible to damage by the shearing forces produced by the blast waves [25,27,28], which primarily affect retinal ganglion cells, as manifested by thinning of the retinal layers and loss of retinal ganglion cell somas [29,30,31]. Furthermore, in rats, blast exposure has been shown to cause visual and retinal changes up to 8 months post-blast, mimicking some of the visual defects seen in human blast-exposed patients [32]. Previous animal studies have evaluated blast-induced changes in retinal and visual function, upregulation of proteins associated with oxidative stress, activation of inflammation, apoptosis, and cell death [25,26,33,34,35,36]. However, to date, no studies have determined the role of melatonin synthesis and signaling in the retina following bTOI. To our knowledge, this is the first study to demonstrate changes in the mRNA levels of genes involved in melatonin synthesis post-blast in the retina in an animal model of bTOI. We also report changes in the mRNA expression of retina-specific proteins, namely *Rhodopsin*, *Melanopsin*, and *RPE65* after blast injury.

Recent studies indicate that melatonin synthesis in the retina shares features with that occurring in the pineal gland, including the same biochemical substrates and enzymatic pathway [37,38]. In the retina, tryptophan hydroxylase (*TPH*), the first enzyme involved in the biosynthesis of serotonin/melatonin, is primarily expressed in the photoreceptor cells and converts tryptophan into 5-hydroxytryptophan, the precursor to serotonin. Hence, an increase or decrease in *TPH* catalytic activity can lead to corresponding alterations in serotonin levels in the retina [39]. Therefore, *TPH* is essential for regulating the circadian rhythm of the eye, which is linked to light–dark cycles [40,41]. Our study noted decreases in *TPH*1 (non-significant) and *TPH*2 mRNA expression levels at 4 h post-blast, followed by an increase in both levels at 24 h and, interestingly, an ensuing decrease at 28 days post-blast. Though no studies have investigated the effect of blast exposure on *TPH* isoforms in the retina, increased mRNA levels of *TPH*2 in the dorsal raphe nucleus at 24 h post-blast have been noted [42,43]. Similarly, transcript levels of *TPH*2 were elevated in the dorsal raphe nucleus acutely post-blast and remained elevated even after one week [44]. Our previous study showed decreased *TPH*1 and increased *TPH*2 mRNA levels at 6 and 24 h post-blast injury in the pineal gland [45]. Several animal studies indicate that *TPH*2 gene expression is modulated by various stressors. For instance, a significant increase in *TPH*2 expression was noted in rat medulla following hypotensive hemorrhage [46]. Similarly, rats exposed to repeated forced swim and chronic variable stress resulted in elevated *TPH*2 expression in the midbrain [47]. Hence, the increased *TPH*1 and *TPH*2 levels in the retina at 24 h post-blast could be at least partly accounted for as a stress response. Interestingly, we noted a decrease in *TPH*1 and *TPH*2 levels in the retina at 28 days post-blast injury. This can be attributed to the loss of retinal ganglion cell axons [48] and subsequent retinal degeneration [25], which have been reported at 4–6 weeks post-blast injury [49].

Melatonin synthesis in the retinal photoreceptors is regulated primarily by changes in the activity of *AANAT*, the key enzyme that converts serotonin to N-acetylserotonin [50]. Apart from playing a role in circadian rhythm, it also plays a key role in neurotransmission and detoxification by acetylating arylalkylamines that may react with retinaldehyde [51]. We have noted a decrease in *AANAT* mRNA expression levels at 24 h and an increase at 28 days post-blast. Furthermore, *ASMT* mRNA levels decreased at 24 h and at 28 days post-blast, indicating perturbed melatonin synthesis in the retina at those time points. In contrast, the mRNA levels of *ASMT* showed an increase at day 7 post-blast. A previous study noted decreased *AANAT* gene expression in the rat frontal cortex at eight months post-blast exposure [52]. Our previous study in rats showed decreased *AANAT* and *ASMT* mRNA levels at 6 h and 24 h post-blast exposure in the pineal gland. The biological importance of time-dependent changes in the mRNA levels of enzymes involved in the melatonin synthesis pathway in the retina needs to be further evaluated.

Serotonin (5-hydroxytryptamine (5-HT)) plays a crucial role in neuroprotection within the eye, particularly in maintaining the health of retinal cells and regulating visual acuity [53,54]. Serotonin enhances retinal cell survival, decreases inflammation, and protects against oxidative damage [55,56]. It acts via serotonin receptors (HTRs) in retinal cells, particularly hydroxytryptamine receptor 1B (HTR1B) and hydroxytryptamine receptor 1D (HTR1D), and it influences the transmission of visual information to the thalamus [57]. Serotonin also regulates glutamate release and receptor activity, minimizing excitotoxicity and preventing damage to retinal neurons [54]. It influences the retinal response to hypoxia, potentially improving tissue oxygenation and decreasing damage caused by ischemia [58]. Serotonin additionally controls blood flow within the retina, regulating blood vessel constriction and dilation [59,60]. Serotonin governs circadian rhythms, the body’s innate biological clocks [61]. The suprachiasmatic nucleus (SCN) in the hypothalamus manages the circadian time system [62]. Serotonin from the raphe nuclei modulates SCN activity, effecting phase shifts and the sleep–wake cycle [63]. It can prevent light-induced phase shifts, sustaining circadian rhythms [62]. This control ensures physiological and behavioral processes are coordinated with the day-night cycle. Hence, serotonin is vital to retinal function and circadian rhythm modulation.

*Rhodopsin* is the most abundant protein in the retina’s rod cells and functions as the primary photoreceptor molecule mediating vision along with the maintenance of circadian rhythm [64,65]. Degeneration of these photoreceptors manifests as an outer nuclear layer thinning, a reduction in electroretinogram amplitudes, and vision loss [66]; hence, studies indicate that *Rhodopsin* can be considered as a biomarker associated with retinal thinning and degeneration [67,68,69]. Furthermore, changes in the *Rhodopsin* levels have been shown to contribute to early events of retinal degeneration [70]. We have noted an increase in mRNA levels of *Rhodopsin* at 24 h and then a decrease at 7 days and 28 days post-blast exposure. Our results align with another study, which showed lighter and diminished staining of *Rhodopsin* in the retina at 7 days and 30 days post-blast exposure [71]. Like Rhodopsin, Melanopsin is a photosensitive protein predominantly located in the retinal ganglion cells, which has also been implicated in regulating circadian rhythm. Repeated blast exposure showed an increase in mRNA levels of *Melanopsin* at 24 h and then a decrease at 7 days and 28 days post-blast. Though there are no reports of a direct link between blast exposure and changes in *Melanopsin* levels, a study by Pérez MP et al. showed high-intensity light exposure of rats induced reduction in *Melanopsin* positive retinal ganglionic cells (RGCs) [72]. In another study, Nadal-Nicolás et al. demonstrated the downregulation of melanopsin^+^ RGCs and melanopsin mRNA level after 7 days post optic nerve crush injury [73]. Moreover, a blast model of mild TBI showed significantly reduced retinal ganglion cells and increased *Melanopsin* immunolabeling in both eyes of C57BL/6 mice after 5 days of blast exposure [74]. Interestingly, studies have shown that *Melanopsin*-expressing retinal ganglion cells (mRGCs) are more resilient to damage from various retinal insults like axonal injury, chronic ocular hypertension, and neurodegeneration compared to other retinal ganglion cell types [75]. Hence, the biological impact of the mRNA expression changes in *Melanopsin* we have seen after blast exposure deserves further investigation.

Melatonin signaling has also been implicated in controlling the daily rhythm of RPE65 protein transcription, thus, regulating the visual cycle [76]. Mutations in the *RPE65* gene are associated with Leber Congenital amaurosis, characterized by progressive retinal photoreceptor cell degeneration and eventually severe vision loss [77]. While direct evidence of the interaction between *RPE65* and blast exposure is limited, blast waves can directly damage the retinal pigment epithelium (RPE), where *RPE65* is primarily located, and bring about changes in the expression levels of *RPE65*. We have noted a decrease in *RPE65* mRNA levels at 4 h, an increase at 24 h, and a decrease at 7 days and 28 days post-blast exposure.

In the retina, the melatonin signaling pathway modulates photoreceptor viability during aging and protects photoreceptors from oxidative stress and apoptosis. Furthermore, melatonin signaling has been shown to regulate retinal dopamine levels, rod/cone electrical coupling, and photopic and scotopic vision [11,37]. Since the retinal pigment epithelium lacks regenerative capacity, slight alterations in melatonin signaling may prompt significant implications for retinal health [78]. Though melatonin or its derivatives are currently not approved by the FDA for the treatment of ocular diseases, several animal studies have indicated the potential benefits of melatonin in addressing such conditions [11]. For instance, melatonin supplementation has been shown to reduce the risk of age-related macular degeneration (AMD). Furthermore, melatonin administration has been shown to protect the retina and delay AMD progression [79]. These findings strongly suggest that a deficiency in melatonin and its signaling pathway(s) may play a role in promoting photoreceptor death following blast-induced eye injury and that melatonin supplementation can be a potential treatment strategy.

In our study, we utilized ferrets as a model to assess blast injury as ferrets’ genome analysis indicates very little genetic divergence from humans. Furthermore, rodents have poor binocular vision, and their visual system is poorly developed compared to higher-order mammals. It is associated with nocturnal activity that is instead primarily dependent upon olfactory and/or auditory perception [80]. The anatomical features of the ocular and central nervous system in monkeys closely resemble those of humans; however, the large number of experimental subjects and associated cost required for experiments of this nature limit their utilization [81,82]. Since ferrets possess developed binocular vision, unlike rodents, we used ferrets as a model for blast-induced eye injury.

Some of the limitations of our study include the following: (1) Melatonin and serotonin levels in the retinal tissue following blast exposure need to be evaluated; (2) protein levels of various enzymes involved in melatonin/serotonin synthesis and retinal specific proteins need to be determined; and (3) functional changes associated with circadian rhythm in the retina post-blast need to be assessed.

## 6. Conclusions

Based upon the importance of melatonin’s pleiotropic effects in the retina, our results appears to demonstrate retinal pathophysiological sequelae triggered by blast exposure. Although our study shows changes in mRNA expression of melatonin synthesis enzymes and photoreceptor genes in the retina at various time points following blast exposure in ferrets, the precise mechanisms that underline the role of melatonin in the retina post-blast exposure remain unclear. Future work investigating the effect of melatonin supplementation to alleviate retinal damage and improve outcomes for individuals affected by blast exposure needs to be evaluated.

## Figures and Tables

**Table 1:** List of primers.

**Gene**	**Forward Primer (5′-3′)**	**Reverse Primer (5′-3′)**
*TPH*1	GCCGATCATCCTGGCTTCAA	CTGCAGGCATGGGTTGGGTA
*TPH*2	ATGCCGACCACCCAGGATTT	AACACGACACCCCACGTCTT
*AANAT*	TCGAGCGCGAAGCCTTCAT	GGTCCCAAAGCGAACCGATG
*ASMT*	ACGACGTACCTGTGTTGGGG	CGCTCACCCTCGGATCTGTA
*Rhodopsin*	GTGGTGGTGTGTAAGCCCAT	CCTCTGGGATGTACCTGGACC
*Melanopsin*	TCTATACCTTCTGCAGGACCAG	CTTATGGAGGCTGCTGACGA
*RPE65*	CCTCTGAATATTGACAAGGCTGAC	ACACGCTTAGGAAAACTCTGAA
*18S rRNA*	GTAACCCGTTGAACCCCATT	CCATCCAATCGGTAGTAGCG

## Data Availability

Data are within the article.

## Disclaimer

Material has been reviewed by the Walter Reed Army Institute of Research. There is no objection to its presentation and/or publication. The opinions or assertions contained herein are the private views of the author, and are not to be construed as official, or as reflecting true views of the Department of the Army or the Department of Defense. The research was conducted under an approved Walter Reed Army Institute of Research (AAALAC-International-accredited) Institutional Animal Care and Use Committee (IACUC) in compliance with the Animal Welfare Act and other federal statutes and regulations relating to animals and experiments involving animals and adheres to principles stated in the Guide for the Care and Use of Laboratory Animals, NRC Publication, 2011 edition.
